# Clinical significance of para‐carinal air cysts in patients with pleuroparenchymal fibroelastosis: The relationship with pneumomediastinum and pneumothorax

**DOI:** 10.1111/crj.13671

**Published:** 2023-07-28

**Authors:** Hideaki Yamakawa, Shintaro Sato, Hiroki Ohta, Makiko Takatsuka, Kenji Kusano, Rie Kawabe, Tomohiro Oba, Keiichi Akasaka, Masako Amano, Jun Araya, Hiroki Sasaki, Hidekazu Matsushima

**Affiliations:** ^1^ Department of Respiratory Medicine Saitama Red Cross Hospital Saitama Japan; ^2^ Department of Respiratory Medicine Tokyo Jikei University Hospital Tokyo Japan; ^3^ Department of Radiology Saitama Cross Hospital Saitama Japan

**Keywords:** para‐carinal air cysts, pleuroparenchymal fibroelastosis, pneumomediastinum, pneumothorax

## Abstract

**Background:**

Para‐tracheal or para‐carinal air cysts (PACs) are often asymptomatic and usually detected incidentally by methods such as computed tomography. Their clinical significance is unclear in patients with pleuroparenchymal fibroelastosis (PPFE).

**Methods:**

We evaluated the clinical significance of PACs in PPFE and their relationship with pneumomediastinum or pneumothorax.

**Results:**

In total, 50 patients had PPFE and 34 (68%) had PACs. Most PACs were para‐carinal (*n* = 30). A para‐tracheal air cyst was detected in only nine patients, which included five patients having both para‐carinal and para‐tracheal air cysts. Overall median survival was 24.7 months. Survival was not significantly different between the patients with [PACs(+)] and without PACs (*P* = 0.268). A high frequency (64%) of the complication of pneumomediastinum or pneumothorax occurred in the overall population during follow‐up. Pneumomediastinum/pneumothorax occurred significantly more frequently in patients with PACs(+) than in those without (76.5% vs. 37.5%; *P* = 0.012). PACs(+) was the only significant risk factor for pneumomediastinum/pneumothorax.

**Conclusions:**

Our data showed that PACs commonly occur in patients with PPFE, and most PACs were para‐carinal air cysts. Additionally, PACs(+) was a significant risk factor for pneumomediastinum/pneumothorax; therefore, clinicians should be more aware of these complications during follow‐up examination, particular in PACs(+) patients with PPFE.

## Graphical abstract

Para‐tracheal or para‐carinal air cysts (PACs) commonly occur in patients with pleuroparenchymal fibroelastosis (PPFE), and most PACs were “para‐carinal air cysts.” PACs was significant risk factor for pneumomediastinum/pneumothorax; therefore, clinicians might be more careful of these complications during follow‐up examination, particular in having PACs patients with PPFE.

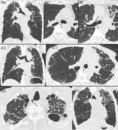

## 1 BRIEF COMMUNICATION

Para‐tracheal or para‐carinal air cysts (PACs) are often asymptomatic and usually detected incidentally by various imaging methods.^1^ Their prevalence reportedly ranges from 0.3% to 6.5%.^1^, ^2^ Some reports stated that PACs occur due to long‐standing increased intraluminal pressure caused by chronic cough or chronic obstructive pulmonary disease (COPD) combined with a weakened tracheal wall,^2^, ^3^, ^4^ but others reported no relation between PAC and COPD. Thus, the relation between PACs and lung disease is controversial,^3^, ^5^ Although most PACs may have no morbid significance, they infrequently lead to pneumomediastinum and pneumothorax,^6^, ^7^ Unlu et al. reported a statistically significant relationship between the presence of para‐tracheal air cysts and upper lobe‐dominant fibrosis and, interestingly, mentioned upper lobe‐dominant fibrosis as a risk factor for para‐tracheal air cysts development.^8^ Pneumomediastinum/pneumothorax were significantly more common in patients with pleuroparenchymal fibroelastosis (PPFE) and were an independent risk factor for this event.^9^, ^10^, ^11^ Therefore, we investigated the clinical significance of PACs in patients with PPFE and their relationship with pneumomediastinum/pneumothorax because, to our knowledge, no published study has focused on these points. We retrospectively screened 59 PPFE patients between April 2018 and March 2022 diagnosed by using previous clinicoradiological criteria.^12^ Each patient's high‐resolution computed tomography (HRCT) scan was reviewed in multi‐disciplinary discussion. In accordance with Lee et al.,^12^ we excluded nine patients because we could not confirm disease progression; thus, 50 PPFE patients were analyzed. PACs are defined as air‐filled structures within para‐tracheal or para‐carinal soft tissue with or without communication within the tracheal‐bronchus lumen and without communication with lung parenchyma and the esophagus.^5^, ^8^ A lower‐lobe interstitial lung disease (ILD) pattern was assessed on HRCT according to the recent idiopathic pulmonary fibrosis guideline.^13^ Categorical baseline characteristics are summarized by frequency and percentage, with continuous characteristics reported as mean ± SD. Fisher's exact test, unpaired *t* test, or Mann–Whitney *U* test was used to detect differences between presence or absence as appropriate. The Kaplan–Meier method and log‐rank test were used to display and compare survival curves of the cohort stratified into two groups. Logistic regression analysis was performed to identify predictive factors associated with complications of pneumomediastinum/pneumothorax during follow‐up. *P* < 0.05 indicated statistical significance. All data were analyzed with SPSS version 22.0 (IBM Japan, Tokyo). Our Institutional Review Board approved this study (21‐W) and waived requirements for informed consent.

**FIGURE 1:**
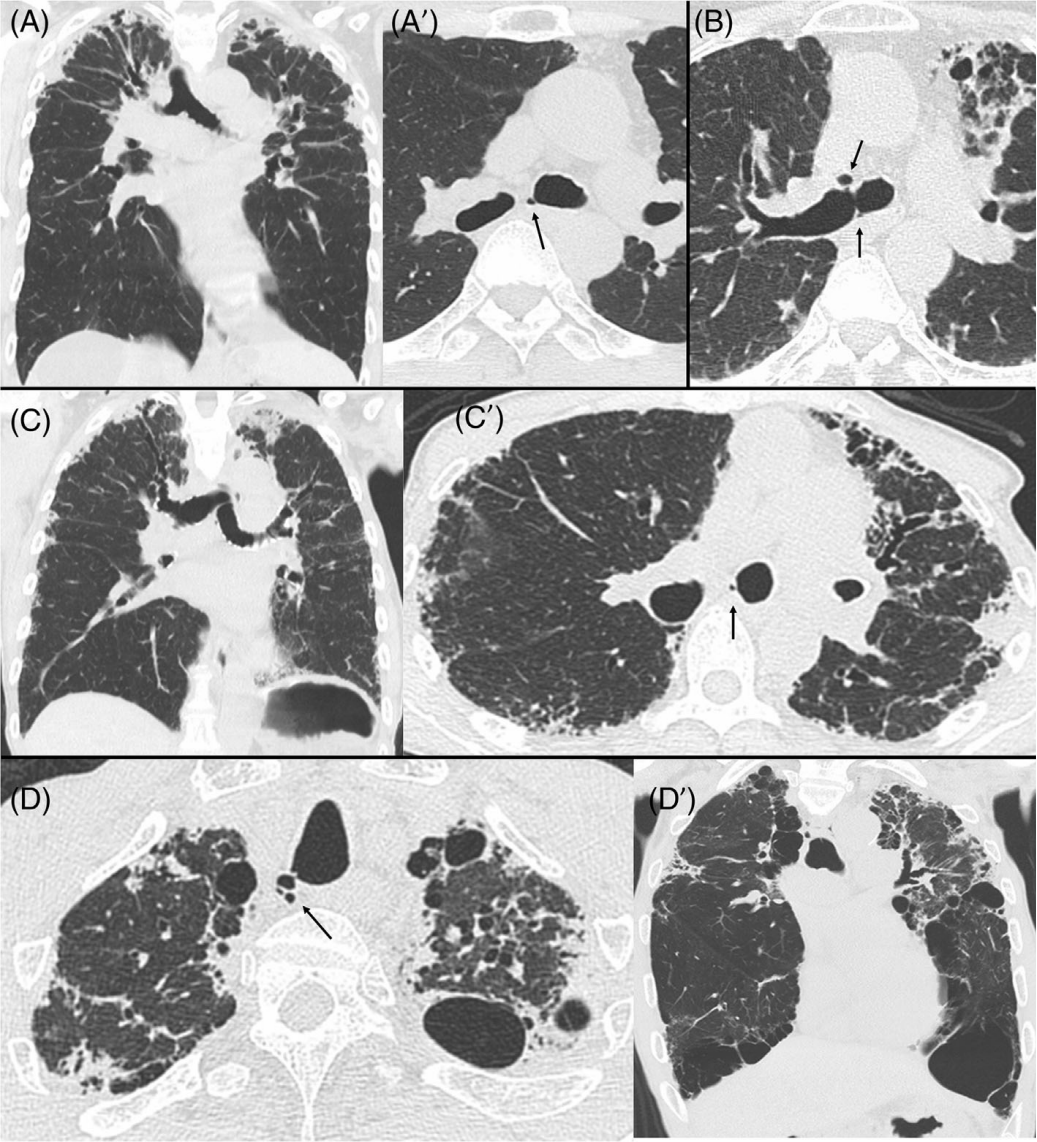
(A/A′) Coronal and axial HRCT images of the chest of a 66‐year‐old woman with PPFE show a small air collection communicating with the left main bronchus (arrow). (B) Chest HRCT image of a 60‐year‐old woman with PPFE shows para‐carinal air cysts in both the right (arrow) and left (arrow) bronchi. (C/C′) Coronal and axial HRCT images of the chest of a 66‐year‐old man show upper lobe‐dominant fibrosis with a para‐carinal air cyst (arrow). (D/D′) Coronal and axial HRCT images of the chest of a 75‐year‐old man with PPFE show a small air collection as a para‐tracheal air cyst (arrow) communicating with the trachea.

In our 50 patients with PPFE, two had rheumatoid arthritis, one had fibrotic hypersensitivity pneumonitis, and the other 47 had idiopathic PPFE. Mean patient age was 73.4 years, 28 (56%) patients were males, and 21 (42%) had a smoking history (Table 1). Median follow‐up time was 18.2 months. Thirty‐four patients (68%) had PACs, most (*n* = 30) in a para‐carinal location (Figure 1). Para‐tracheal air cysts were detected in only nine patients, including five patients with both types. The location of PACs was as follows: right side in 11 patients, left side in 17 patients, and both sides of the trachea‐bronchus in six patients.

**TABLE 1:** Patient characteristics (*n* = 50).

Characteristic	No. of patients (total)	PACs(+) (*n* = 34)	PACs(−) (*N* = 16)	*P* value
Male, *n* (%)	28 (56.0)	17 (50.0)	11 (68.8)	0.240
Age (years), mean ± SD	73.4 ± 7.2	72.9 ± 7.2	74.6 ± 7.4	0.433
Smoking history, *n* (%)	21 (42.0)	12 (35.3)	9 (56.3)	0.222
Cough, *n* (%)	29 (58.0)	22 (64.7)	7 (43.8)	0.222
Dyspnea on exertion, *n* (%)	43 (86.0)	32 (94.1)	11 (68.8)	0.027
Pulmonary emphysema, *n* (%)	2 (4.0)	1 (2.9)	1 (6.3)	0.542
BMI (kg/m^2^), mean ± SD	18.0 ± 2.9	17.9 ± 2.7	18.3 ± 3.3	0.676
%FVC, mean ± SD	70.0 ± 18.2	70.7 ± 18.1	68.8 ± 19.1	0.776
%DL_CO_, mean ± SD	92.7 ± 24.6	96.2 ± 24.7	85.1 ± 23.5	0.218
RV/TLC (%), mean ± SD	46.6 ± 9.8	48.5 ± 9.3	42.4 ± 9.9	0.076
Respiratory complication during follow‐up, *n* (%)				
Pneumomediastinum and/or pneumothorax	32 (64.0)	26 (76.5)	6 (37.5)	0.012
Pneumomediastinum	7 (14.0)	7 (20.6)	0 (0.0)	0.081
Pneumothorax	10 (20.0)	6 (17.6)	4 (25.0)	0.707
Both pneumomediastinum and pneumothorax	15 (30.0)	13 (38.2)	2 (12.5)	0.099
Respiratory infection such as NTM and *Aspergillus*	8 (16.0)	6 (17.6)	2 (12.5)	>0.999
Lower‐lobe ILD pattern, *n* (%)				
UIP	27 (54.0)	16 (47.1)	11 (68.8)	0.339
Non‐UIP	7 (14.0)	5 (14.7)	2 (12.5)	
None	16 (32.0)	13 (38.2)	3 (18.8)	
Medication for ILD during follow‐up, *n* (%)				
Anti‐inflammatory agent	7 (14.0)	4 (11.8)	3 (18.8)	0.666
Anti‐fibrotic agent	9 (18.0)	4 (11.8)	5 (31.2)	0.124
Total deaths during follow‐up, *n* (%)	28 (56.0)	21 (61.8)	7 (43.8)	0.360
Acute exacerbation of ILD	1	1	0	0.452
Chronic disease progression	23	18	5	
Pulmonary infection	4	2	2	
Median follow‐up, months (range)	18.2 (2.5–53.1)	17.5 (2.5–53.1)	28.1 (3.6–52.7)	0.417

Abbreviations: BMI, body mass index; DL_CO_, diffusing capacity for the lung carbon monoxide; FVC, forced vital capacity; ILD, interstitial lung disease; NTM, non‐tuberculous mycobacteria; PACs, para‐tracheal or para‐carinal air cysts; RV, residual volume; SD, standard deviation; TLC, total lung capacity; UIP, usual interstitial pneumonia.

Dyspnea on exertion was significantly more frequent in the PACs(+) than PACs(−) group (Table 1). No significant differences were observed between the two groups in sex, age, smoking history, radiological emphysema, body mass index, pulmonary function, complications of respiratory infection, lower‐lobe ILD pattern, ILD medications, and cause of death. Log‐rank tests showed no differences in survival between the two groups (*P* = 0.268). Overall median survival was 24.7 months. Five‐year survival rates were 20.8% in the PACs(+) group and 54.1% in the PACs(−) group.

During follow‐up, complications of pneumomediastinum/pneumothorax occurred highly frequently (64%) among all patients and significantly more frequently in the PACs(+) versus PACs(−) group (76.5% vs. 37.5%; *P* = 0.012) (Table 1). Among the patients with complications of pneumomediastinum/pneumothorax, in the PACs(+) group (*n* = 26), seven patients had pneumomediastinum only, six had pneumothorax only, and 13 had both pneumomediastinum and pneumothorax. Contrastingly, in the PACs(−) group (*n* = 6), four patients had pneumothorax only, two had both pneumomediastinum and pneumothorax, and none had pneumomediastinum only. PACs(+) was the only significant risk factor for pneumomediastinum/pneumothorax (odds ratio: 7.2, 95% confidence interval: 1.311–39.557; *P* = 0.023) by multivariate analysis with forward variable selection (Table 2). Among the patients with complications of pneumothorax, in the PACs(+) group (*n* = 19; six patients with pneumothorax only/13 patients with pneumomediastinum and pneumothorax), in the six patients with PACs on the right side, the pneumothorax was on the right side in one, left side in two, and both sides in three patients. In the eight patients with PACs on the left side, the pneumothorax was on the right side in one, left side in four, and both sides in three patients. In the patients with bilateral PACs (*n* = 5), the pneumothorax was on the right side in one, left side in four, and both sides in none.

**TABLE 2:** Risk factors of pneumomediastinum or pneumothorax with PPFE.

	Univariate analysis				Multivariate analysis		
	*n*	OR	95% CI	*P* value	OR	95% CI	*P* value
PACs (+)	50	5.417	1.498, 19.588	**0.010**	7.200	1.311, 39.557	**0.023**
Male	50	0.721	0.223, 2.335	0.586	‐		
Age	50	0.965	0.886, 1.051	0.413	‐		
Smoking history	50	0.600	0.186, 1.931	0.392	‐		
Cough	50	2.386	0.732, 7.780	0.149	‐		
Dyspnea on exertion	50	15.500	1.684, 142.628	**0.016**	n.c.		
Pulmonary emphysema	50	n.c.			‐		
BMI	50	0.802	0.641, 1.003	0.053	‐		
%FVC	38	0.949	0.907, 0.992	**0.021**	n.e.		
%DL_CO_	35	0.995	0.967, 1.022	0.698	‐		
RV/TLC	37	1.096	1.006, 1.195	**0.036**	n.e.		
Lower‐lobe ILD pattern	50				‐		
UIP	27	1.556	0.436, 5.546	0.496			
Non‐UIP	7	1.944	0.287, 13.188	0.496			
None	16	1.000	ref				

*Note*: Bold values denote statistical significance at the *P* < 0.05 level.

Abbreviations: BMI, body mass index; CI, confidence interval; DL_CO_, diffusing capacity for the lung carbon monoxide; FVC, forced vital capacity; ILD, interstitial lung disease; n.c., not calculated; n.e., not estimated; OR, odds ratio; PACs, para‐tracheal or para‐carinal air cysts; PPFE; pleuroparenchymal fibroelastosis; RV, residual volume; TLC, total lung capacity; UIP, usual interstitial pneumonia.

To our knowledge, this is the first report on the frequency and significance of PACs in patients with PPFE. Surprisingly, the prevalence of PACs, which are common in PPFE patients, was 68%. Moreover, although PACs are generally located 4–5 cm below the vocal cords as para‐tracheal air cysts, most PACs in our patients were para‐carinal air cysts. A previous study of PACs and PPFE by Unlu et al.^8^ showed most PACs (98.9%) to be located at the right posterolateral aspect of the trachea as para‐tracheal air cysts and not as para‐carinal air cysts. Although the reason for this discrepancy is unclear, the patient populations were considerably different: Unlu et al. did not narrow their study to patients with PPFE, whereas the present study did. Moreover, Higuchi et al. reported that para‐carinal air cysts may be a relatively common (41%) CT finding regardless of whether pulmonary disorders exist.^5^ Increased tracheal intraluminal pressure caused by chronic cough or obstructive ventilatory failure combined with weakened trachea‐bronchus wall musculature from repeated respiratory infections can lead to the acquired form of PACs.^14^ The high frequency of PACs in our PPFE cohort may be compatible with compensatory increased end‐expiratory air trapping of the lower lung against shrinking of the upper lung by PPFE.^11^

Pneumomediastinum/pneumothorax is common in PPFE patients,^9^, ^10^, ^11^ and >60% of our patients had these complications. PACs was the only significant risk factor for pneumomediastinum/pneumothorax, suggesting a strong association between PACs and the risk for pneumomediastinum/pneumothorax in PPFE patients. Pneumomediastinum/pneumothorax in patients with PPFE may be caused by low pleural resistance to shear stress or by cysts in the apical fibrotic area.^15^, ^16^, ^17^ If PACs cause pneumomediastinum/pneumothorax in PPFE patients, pneumomediastinum may be more common than pneumothorax for anatomical reasons. In the PPFE patients complicated by pneumothorax, pneumomediastinum co‐occurred in 13 of the 19 PACs(+) patients in our cohort, but the complication of pneumomediastinum only occurred in seven patients in the PACs(+) group and in none in the PACs(−) group. Clearly, PACs(+) patients with PPFE were more likely to suffer the complication of pneumomediastinum.

We often experience patients with repeated recurrence of pneumothorax from PPFE, even if their pneumothorax was once healed by surgical therapy. The relationship between the location of PACs and the direction of pneumothorax was not relevant in our study. Although some reports showed that PACs can lead to pneumomediastinum/pneumothorax,^6^, ^7^ we speculate that both lung parenchyma and PACs were complexly intertwined in the development of pneumomediastinum/pneumothorax in the PPFE patients. As well, as reported by Kono et al.,^18^ corticosteroid use can potentially cause the development of pneumothorax, but we could not clearly determine whether treatment for ILD itself impacted pneumomediastinum/pneumothorax and, as such, the natural disease course.

If PACs are associated with the development of pneumomediastinum/pneumothorax in PPFE patients, PACs may be likely to increase over time. We previously reported enlargement of PACs over 5 years in a PPFE patient.^19^ However, we could not find cases with new development of PACs during the follow‐up period. Although we cannot state this definitively, longer, more careful observation may be required in these patients.

In conclusion, PACs were common in the PPFE patients, and most were para‐carinal air cysts. PACs were a significant risk factor for pneumomediastinum/pneumothorax, so clinicians should be aware of these complication during follow‐up, particularly in PACs(+) patients with PPFE. As limitations, this retrospective study conducted in a single referral hospital limits the generalizability of our findings, and accurate conclusions cannot be drawn due to the small number of patients. Further studies with larger sample size are needed to clarify the significance of PACs in patients with PPFE.

## AUTHOR CONTRIBUTIONS

Hideaki Yamakawa, Shintaro Sato, and Hiroki Ohta acquired the data; Hideaki Yamakawa, Shintaro Sato, Hiroki Ohta, Makiko Takatsuka, Kenji Kusano, Rie Kawabe, Tomohiro Oba, Keiichi Akasaka, Masako Amano, Jun Araya, Hiroki Sasaki, and Hidekazu Matsushima analyzed and interpreted the clinical data; and Hideaki Yamakawa and Hidekazu Matsushima drafted the manuscript. All authors read and approved the final manuscript.

## CONFLICT OF INTEREST STATEMENT

All authors declare no conflicts of interest.

## ETHICS STATEMENT

The current study got ethical approval from the Saitama Red Cross Hospital Research Ethics Board and did not need informed consent. The ethics number is 21‐W.

## Data Availability

Data sharing is not applicable to this article as no new data were created or analyzed in this study.
